# Evaluation of gene-environment interactions for colorectal cancer susceptibility loci using case-only and case-control designs

**DOI:** 10.1186/s12885-019-6456-9

**Published:** 2019-12-18

**Authors:** Nan Song, Jeeyoo Lee, Sooyoung Cho, Jeongseon Kim, Jae Hwan Oh, Aesun Shin

**Affiliations:** 1 Cancer Research Institute, Seoul National University, Seoul, South Korea; 2 Department of Epidemiology and Cancer Control, St. Jude Children’s Research Hospital, Memphis, TN USA; 3 Department of Preventive Medicine, College of Medicine, Seoul National University, Seoul, South Korea; 4 Molecular Epidemiology Branch, National Cancer Center, Goyang, South Korea; 5 Center for Colorectal Cancer, National Cancer Center, Goyang, South Korea

**Keywords:** Colorectal cancer, Gene-environment interaction, Single-nucleotide polymorphism, Case-only design, Case-control design

## Abstract

**Background:**

Genome-wide association studies (GWAS) have identified more than 40 colorectal cancer susceptibility loci, but only a small fraction of heritability was explained. To account for missing heritability, we investigated gene-environment interactions (G × Es) between GWAS-identified single-nucleotide polymorphisms (SNPs) and established risk or protective factors for colorectal cancer using both case-only and case-control study designs.

**Methods:**

Data on 703 colorectal cancer cases and 1406 healthy controls from the National Cancer Center in Korea were used. We tested interactions between 31 GWAS-identified SNPs and 13 established risk or protective factors for colorectal cancer (family history, body mass index, history of colorectal polyps, inflammatory bowel disease, and diabetes mellitus, alcohol drinking, smoking, regular exercise, regular aspirin use, postmenopausal hormone replace therapy, red meat and processed meat intake, and dairy consumption). Logistic regression models were used to assess G × Es for colorectal cancer risk.

**Results:**

The SNP rs4444235 at 14q22.2 interacted with regular exercise in colorectal cancer (*p*_case-only_ = 2.4 × 10^− 3^, *p*_case-control_ = 1.5 × 10^− 3^). The risk allele (C) of rs4444235 increased the risk of colorectal cancer in regularly exercising individuals (OR = 1.47, 95% CI = 1.02–2.10) but decreased the risk in non-exercising individuals (OR = 0.76, 95% CI = 0.62–0.94). Furthermore, the G × E between the SNP rs2423279 at 20p12.3 and regular aspirin use was statistically significant (*p*_case-only_ = 7.7 × 10^− 3^, *p*_case-control_ = 1.6 × 10^− 3^). The additive effect of the risk allele (T) of rs2423279 on colorectal cancer risk was increased among regular aspirin users (OR = 4.62, 95% CI = 1.97–10.80).

**Conclusion:**

Our results suggest that SNP rs4444235 at 14q22.2 and SNP rs2423279 at 20p12.3 may interact with regular exercise and aspirin use in colorectal carcinogenesis.

## Background

The genetic heritability for colorectal cancer was approximately 35% (95% confidence interval (CI) = 10–48%) in a twin study [1]. Furthermore, common single-nucleotide polymorphisms (SNPs) were expected to explain at least 7.4% of the heritability [2]. Although genome-wide association studies (GWASs) have identified more than 40 genetic susceptibility regions related to colorectal cancer risk with a nominal genome-wide significance threshold (*p*-value = 5 × 10^− 8^) [3], the common SNPs discovered by previous GWAS only accounted for 0.65% of the heritability of colorectal cancer, resulting in remaining missing heritability [2]. Accordingly, gene-environment interactions (G × Es) were suggested to contribute to the missing heritability [4]. Furthermore, since GWAS-identified SNPs might be located on non-coding regions or unknown genes of the DNA since due to a non-hypothesis-driven approach of GWAS, elucidation G × Es may allow a a better understanding of the biological mechanism of the genetic variations [5].

To investigate the potential contribution of G × Es to colorectal cancer, several studies have evaluated G × Es for colorectal cancer susceptibility loci identified by previous GWAS [6–13] and at a genome-wide level [14]. Most studies have adopted a case-control study design to study G × Es [6–12], which has the advantage of being relatively robust and maintaining a desired type I error rate [15]. Few studies on G × Es have used a case-only design for colorectal cancer [13, 14]. Although the case-only design on G × Es is considered an alternative to case-control design due to potential false positives by unverified assumption of independence between genetic and environmental factors, it allows for more efficient estimation and more powerful association tests to be performed on G × Es than case-control design [16].

For robust and powerful detection, we used both case-only and case-control approaches to investigate G × Es for colorectal cancer. We focused on 31 SNPs in colorectal cancer susceptibility loci identified by previous GWAS and 13 established environmental risk or protective factors.

## Methods

### Study population

The study population was recruited from the National Cancer Center (NCC) in Korea as previously reported [11–13] and presented in Additional file 1: Figure S1. In brief, among 1427 incident colorectal cancer cases who were diagnosed and had a surgery between 2010 and 2013, 1070 cases agreed to participate in the study. We excluded patients who did not complete questionnaires or patients whose blood samples were not insufficient for genotyping. Thus, a total of 703 colorectal cancer patients were included in the analyses. The 14201 healthy controls were recruited among people who underwent a health screening examination, which was a benefit program of the National Health Insurance between 2007 and 2014. Among 9037 people who consented to participate in the study and completed the questionnaire, a total of 1406 healthy controls were included in the analyses by 1:2 frequency matching on 5-year age and sex. All study participants provided written informed consent, and the study was approved by the institutional review board (IRB) of the NCC (IRB No. NCCNCS-10-350 and NCC 2015–0202).

### Data collection

In this study, the established colorectal cancer risk or protective factors were defined as factors or interventions with adequate evidence of increased or decreased risk of colorectal cancer based on the latest Physician Data Query (PDQ®) cancer information summaries on colorectal cancer prevention of the National Cancer Institute (NCI) updated by Mar 1, 2018 [17] and the Colorectal Cancer Facts & Figs. 2017–2019 of American Cancer Society (ACS®) [18]. Accordingly, the environmental factors considered in this analysis included family history of colorectal cancer, BMI, history of colorectal polyps, history of inflammatory bowel disease (IBD), history of DM, postmenopausal HRT, red meat intake, processed meat intake, and dairy consumption. Milk consumption was excluded in this analysis due to substantial overlap with diary product consumption.

The data for the selected environmental factors were collected from the structured questionnaires composed of two parts: one was concerned with demographic and epidemiological factors, described in detail elsewhere [11], and the other was a semiquantitative food frequency questionnaire (SQFFQ) [19]. Face-to-face interviews were conducted for colorectal cancer patients by trained interviewers using the written questionnaires. Controls completed the questionnaires themselves and their responses were validated by telephone interviews. The questionnaires were developed based on Korean National Health and Nutrition Examination survey, where internal quality assurance as well as external quality control program were managed by the Korea Centers for Disease Control and Prevention [20].

### SNP selection and genotyping

We included 31 SNPs previously identified to be associated with colorectal cancer risk with nominal genome-wide statistical significance (*p*-value < 5 × 10^− 8^) as described previously in detail [11–13]. DNA extraction and genotyping were performed on BioRobot M48 automatic extraction equipment with the MagAttract DNA Blood M48 Kit (Qiagen, Hilden, Germany) and an Agenabio MassArray iPLEX® gold assay (Agena Bioscience, Inc., San Diego, CA, US). Briefly, the genotype data for any SNPs were excluded according to the quality control procedures for the following reasons: genotyping failure, monomorphic or minor allele frequency (MAF) < 0.01, or *p*-value for deviations from Hardy-Weinberg equilibrium (HWE) < 0.01 in controls.

### Statistical analysis

To test the difference in the distribution of environmental factors between colorectal cancer cases and controls, a chi-square test for categorical variables and a T-test for continuous variables were conducted. The environmental factors were dichotomized, and a category known to be a lower risk group for colorectal cancer was considered as a reference. For genetic factors, individual SNP alleles were designated as risk or effect alleles based on the literature review. Based on each genotype of SNPs coded as 0, 1, or 2 copies of risk or effect alleles, we calculated MAF, risk or effect allele frequency (RAF), and *p*-value for deviations from HWE in controls. To assess the effects of the environmental factors and genetic factors assuming a log-additive model on colorectal cancer risk, a logistic regression model was used.

To detect the statistical significance of G × Es, we employed both case-only and case-control designs. In the case-only analysis, each SNP genotype was treated as an independent variable, and each status of dichotomized environmental factors was treated as a dependent variable using a logistic regression model. Under the same setting, control-only analysis was also conducted to test the assumption of independence between genetic and environmental factors. In case-control logistic analysis on colorectal cancer risk, independent variables included not only the SNP genotype and binary status of environmental factors but also the meaning of G × E terms of those genetic and environmental factors. To be eligible for further analysis, the SNPs for which the nominal *p*-value for G × E was < 0.05 in both case-only and case-control analyses and at least one *p*-value for G × E was < 1.61 × 10^− 3^ (Bonferroni-corrected *p*-value; 0.05/31) to account for multiple testing were selected. To evaluate the genetic effects on colorectal cancer risk that were modified by environmental factors, association tests for the selected SNPs with statistically significant *p*-values for G × E were conducted, stratified by corresponding environmental factor status. For those SNPs, we estimated effects for each genotype as well as effects assuming log-additive, dominant, and recessive models.

The logistic models that only included genetic variables were unadjusted. If models included environmental variables, all analyses were adjusted for age and sex. Potential confounders were chosen based on an association test between environmental factors and colorectal cancer risk. To prevent a problem of multicollinearity among the potential confounders, if a statistically significant correlation was observed between any two paired variables, the variable making a smaller contribution to colorectal cancer risk was dropped. Accordingly, analyses were adjusted for age, sex, family history of colorectal cancer, history of DM, regular exercise, and dairy consumption. Moreover, the dietary factor values were adjusted for total energy intake using the residual method as described elsewhere [21]. All associations and statistical significance were estimated by odds ratio (OR), 95% CI, and two-sided *p*-value using SAS 9.4 software (SAS Institute, Inc., Cary, NC, US).

## Results

Table 1 shows the characteristics of the study population and their associations with colorectal cancer risk. Our study population consisted of 703 colorectal cancer cases and 1406 healthy controls. Given that cases and controls were frequency-matched by age and sex, they had a similar mean age (56.4 years in cases and 56.0 years in controls) and the same distribution of sex (31.7% women and 68.3% men). We observed statistically significant differences in family history of colorectal cancer, BMI, history of DM, regular exercise, regular aspirin use, postmenopausal HRT use, red and processed meat intake, and dairy product consumption (*P* < 0.05). After adjustment for covariates, we observed a statistically significant association for an increased risk of colorectal cancer with family history of colorectal cancer (OR = 2.27, 95% CI = 1.56–3.32, *P* < 0.01), history of DM (OR = 2.27, 95% CI = 1.56–3.32, *P* < 0.01), nonregular exercise (OR = 2.97, 95% CI = 2.43–3.62, *P* < 0.01), nonregular aspirin use (OR = 3.26, 95% CI = 1.97–5.41, *P* < 0.01), and dairy consumption less than 400 g/day (OR = 2.23, 95% CI = 1.53–3.25, *P* < 0.01). Contrary to previous studies, we observed a statistically significant association for a decreased risk of colorectal cancer with red meat intake equal to or greater than 100 g/day (OR = 0.66, 95% CI = 0.47–0.92, *P* = 0.02).

**Table 1:** Characteristics of colorectal cancer cases and controls and associations with colorectal cancer risk

Characteristics	Colorectal cancer cases		Controls		*P*^a^	OR	(95% CI)^b^	*P*^b^
	(*N* = 703, 100%)		(*N* = 1406, 100%)					
	N	(%)	N	(%)				
Age, years								
mean (SD)	56.4	(9.6)	56.0	(9.1)	0.31			
< 50	159	(22.6)	318	(22.6)	> 0.99	1.00	(ref.)	
≥ 50	544	(77.4)	1088	(77.4)		1.10	(0.87–1.40)	0.41
Sex								
Women	223	(31.7)	446	(31.7)	> 0.99	1.00	(ref.)	
Men	480	(68.3)	960	(68.3)		0.96	(0.78–1.18)	0.68
Family history of colorectal cancer								
No	636	(90.5)	1339	(95.2)	< 0.01	1.00	(ref.)	
Yes	67	(9.5)	67	(4.8)		2.27	(1.56–3.32)	< 0.01
BMI, kg/m^2^								
mean (SD)	23.8	(3.4)	24.1	(2.7)	0.04			
< 25.0	478	(68.0)	930	(66.2)	0.47	1.00	(ref.)	
≥ 25.0	225	(32.0)	470	(33.4)		0.87	(0.71–1.07)	0.19
History of colorectal polyps								
No	628	(89.3)	1227	(87.3)	0.17	1.00	(ref.)	
Yes	75	(10.7)	179	(12.7)		0.94	(0.69–1.28)	0.70
History of IBD								
No	701	(99.7)	1401	(99.6)	0.79	1.00	(ref.)	
Yes	2	(0.3)	5	(0.4)		0.67	(0.12–3.87)	0.66
History of DM								
No	616	(87.6)	1287	(91.5)	< 0.01	1.00	(ref.)	
Yes	87	(12.4)	119	(8.5)		2.27	(1.56–3.32)	< 0.01
Alcohol drinking								
Never	212	(30.2)	419	(29.8)	0.87	1.00	(ref.)	
Ever	491	(69.8)	987	(70.2)		1.07	(0.84–1.36)	0.59
Smoking								
Never	316	(45.0)	617	(43.9)	0.64	1.00	(ref.)	
Ever	387	(55.1)	789	(56.1)		0.88	(0.68–1.15)	0.36
Regular exercise								
Yes	229	(32.6)	566	(40.3)	< 0.01	1.00	(ref.)	
No	474	(67.4)	833	(59.3)		2.97	(2.43–3.62)	< 0.01
Regular aspirin use								
Yes	20	(2.8)	134	(9.5)	< 0.01	1.00	(ref.)	
No	683	(97.2)	1272	(90.5)		3.26	(1.97–5.41)	< 0.01
HRT in postmenopausal women								
Ever	31	(19.1)	99	(30.9)	< 0.01	1.00	(ref.)	
Never	130	(80.3)	219	(68.4)		1.50	(0.92–2.44)	0.11
Red meat intake, g/day^c^								
mean (SD)	51.2	(33.4)	58.0	(39.0)	< 0.01			
< 100	643	(87.3)	1228	(87.3)	< 0.01	1.00	(ref.)	
≥ 100	58	(8.3)	176	(12.5)		0.66	(0.47–0.92)	0.02
Processed meat intake, g/day^c^								
mean (SD)	1.1	(9.1)	2.5	(13.2)	< 0.01			
< 50	699	(99.4)	1395	(99.2)	0.29	1.00	(ref.)	
≥ 50	2	(0.3)	9	(0.6)		0.78	(0.16–3.93)	0.77
Dairy consumption, g/day^c^								
mean (SD)	72.6	(147.5)	236.4	(807.3)	< 0.01			
≥ 400	16	(2.3)	198	(14.1)	< 0.01	1.00	(ref.)	
< 400	685	(97.8)	1206	(85.8)		2.23	(1.53–3.25)	< 0.01

*SD* standard deviation, *BMI* body mass index, *IBD* inflammatory bowel disease, *DM* diabetes mellitus, *HRT* hormone replacement therapy, *OR* odds ratio, *CI* confidence interval

^a^Chi-square test for categorical variables and T-test for continuous variables

^b^Logistic regression analysis adjusted for age, sex, family history of colorectal cancer, history of DM, regular exercise, and dairy consumption

^c^Dietary factor values were adjusted for total energy intake using the residual method

Table 2 shows the associations between susceptibility SNPs and colorectal cancer risk in previously published GWAS and the current study. Among 31 previously reported SNPs, 13 SNPs (rs647161 at 5q31.1, rs6983267 at 8q24.21, rs7014346 at 8q24.21, rs10505477 at 8q24.21, rs10795668 at 10p14, rs704017 at 10q22.3, rs11196172 at 10q25.2, rs174537 at 11q12.2, rs174550 at 11q12.2, rs1535 at 11q12.2, rs4779584 at 15q13.3, rs10411210 at 19q13.11, and rs2423279 at 20p12.3) showed statistical evidence of association with colorectal cancer risk in the same direction as previous results, with nominal *p*-values ranging from 0.05 to 2.0 × 10^− 4^. The remaining 10 SNPs (rs3802842 at 11q23.1, rs10849432 at 12p13.31, rs10774214 at 12p13.32, rs7136702 at 12q13.13, rs4444235 at 14q22.2, rs4939827 at 18q21.1, rs1800469 at 19q13.2, rs2241714 at 19q13.2, rs961253 at 20p12.3, rs4813802 at 20p12.3) of the 18 SNPs showed evidence of association in the same direction even though it was not statistically significant.

**Table 2:** Associations between susceptibility SNPs and colorectal cancer risk in previously published GWAS and current study

SNP	Chromosomal region	Mapped gene^a^	Allele^b^		Previously published study					Current study				
					RAF^c^	OR	(95% CI)^d^	*P*^d^	Reference (study population)	RAF^c^	*P*_HWE_^c^	OR	(95% CI)^d^	*P*^d^
			A1	A2										
rs6687758	1q41	*intergenic*	G	A	0.20–0.28	1.09	(1.06–1.12)	2.3 × 10^−09^	Houlston et al. Nat Genet. 2010 (European)	0.27	0.43	1.14	(0.98–1.33)	0.09
rs10936599	3q26.2	*MYNN*	T	C	0.20–0.26	0.93	(0.91–0.96)	3.4 × 10^−08^	Houlston et al. Nat Genet. 2010 (European)	0.61	0.22	1.02	(0.89–1.16)	0.78
rs647161	5q31.1	*C5orf66*	A	C	0.31	1.17	(1.11–1.22)	3.8 × 10^−10^	Jia et al. Nat Genet. 2012 (East Asian)	0.29	0.94	1.22	(1.09–1.45)	1.5 × 10^−03^
rs7758229	6q25.3	*SLC22A3*	T	G	0.22	1.28	(1.18–1.39)	7.9 × 10^−09^	Cui et al. Gut. 2011 (East Asian)	0.21	0.14	1.08	(0.92–1.26)	0.37
rs6983267	8q24.21	*CASC8, CCAT2*	G	T	0.49	1.21	(1.15–1.27)	1.3 × 10^− 14^	Tomlinson et al. Nat Genet. 2007 (European)	0.43	0.22	1.20	(1.06–1.37)	5.0 × 10^−03^
rs7014346	8q24.21	*CASC8*	A	G	0.18	1.19	(1.15–1.23)	8.6 × 10^−26^	Tenesa et al. Nat Genet. 2008 (European)	0.29	0.79	1.16	(1.01–1.34)	0.03
rs10505477	8q24.21	*CASC8*	A	G	0.50	1.17	(1.12–1.23)	3.2 × 10^−11^	Zanke et al. Nat Genet. 2007 (European)	0.42	0.16	1.21	(1.07–1.38)	3.5 × 10^−03^
rs10795668	10p14	*LOC105376400*	A	G	0.33	0.89	(0.86–0.91)	2.5 × 10^−13^	Tomlinson et al. Nat Genet. 2008 (European)	0.39	0.73	0.85	(0.74–0.97)	0.02
rs704017	10q22.3	*ZMIZ1-AS1*	G	A	0.32	1.10	(1.06–1.13)	2.1 × 10^−08^	Zhang et al. Nat Genet. 2014 (East Asian)	0.34	0.38	1.17	(1.02–1.34)	0.02
rs11196172	10q25.2	*TCF7L2*	A	G	0.68	1.14	(1.10–1.18)	1.0 × 10^−12^	Zhang et al. Nat Genet. 2014 (East Asian)	0.73	0.52	1.21	(1.04–1.41)	0.01
rs1665650	10q26.2	*HSPA12A*	T	C	0.32	1.13	(1.08–1.19)	8.6 × 10^−07^	Jia et al. Nat Genet. 2012 (East Asian)	0.33	0.13	0.94	(0.81–1.08)	0.38
rs174537	11q12.2	*MYRF*	G	T	0.59	1.16	(1.12–1.19)	9.2 × 10^−21^	Zhang et al. Nat Genet. 2014 (East Asian)	0.67	0.72	1.23	(1.07–1.42)	4.1 × 10^−03^
rs174550	11q12.2	*FADS1*	T	C	0.59	1.15	(1.12–1.19)	1.6 × 10^−19^	Zhang et al. Nat Genet. 2014 (East Asian)	0.67	0.67	1.19	(1.04–1.37)	0.01
rs1535	11q12.2	*FADS2*	A	G	0.59	1.15	(1.12–1.19)	8.2 × 10^−20^	Zhang et al. Nat Genet. 2014 (East Asian)	0.68	0.86	1.20	(1.04–1.38)	0.01
rs3802842	11q23.1	*COLCA1, COLCA2*	C	A	0.43	1.11	(1.08–1.15)	5.8 × 10^− 10^	Tenesa et al. Nat Genet. 2008 (East Asian)	0.41	0.13	1.02	(0.89–1.16)	0.79
rs10849432	12p13.31	*intergenic*	T	C	0.82	1.14	(1.09–1.18)	5.8 × 10^− 10^	Zhang et al. Nat Genet. 2014 (East Asian)	0.82	0.66	1.13	(0.95–1.34)	0.17
rs10774214	12p13.32	*CCND2-AS1*	T	C	0.35	1.17	(1.11–1.23)	5.5 × 10^−10^	Jia et al. Nat Genet. 2012 (East Asian)	0.42	0.84	1.08	(0.95–1.23)	0.25
rs11169552	12q13.13	*ATF1, LOC105369765*	T	C	0.23–0.26	0.92	(0.90–0.95)	1.9 × 10^−10^	Houlston et al. Nat Genet. 2010 (European)	0.33	0.51	1.03	(0.90–1.18)	0.71
rs7136702	12q13.13	*intergenic*	T	C	0.32–0.39	1.06	(1.04–1.08)	4.0 × 10^−08^	Houlston et al. Nat Genet. 2010 (European)	0.52	0.04	1.03	(0.90–1.18)	0.65
rs4444235	14q22.2	*intergenic*	C	T	0.46	1.11	(1.08–1.15)	8.1 × 10^−10^	Houlston et al. Nat Genet. 2008 (European)	0.52	0.52	1.02	(0.90–1.15)	0.80
rs1957636	14q22.3	*LOC105370507*	A	G	0.38–0.43	1.08	(1.06–1.11)	1.4 × 10^−09^	Tomlinson et al. PLoS Genet. 2011 (European)	0.60	0.74	0.90	(0.79–1.02)	0.11
rs4779584	15q13.3	*intergenic*	A^e^	G^e^	0.19	1.18	(1.11–1.24)	1.8 × 10^−08^	Peters et al. Hum Genet. 2011 (European)	0.84	0.91	1.21	(1.00–1.46)	0.05
rs9929218	16q22.1	*CDH1*	A^e^	G^e^	0.29	0.91	(0.89–0.94)	1.2 × 10^−08^	Houlston et al. Nat Genet. 2008 (European)	0.15	0.47	1.11	(0.93–1.33)	0.24
rs12603526	17p13.3	*NXN*	C	T	0.30	1.10	(1.06–1.14)	3.4 × 10^−08^	Zhang et al. Nat Genet. 2014 (East Asian)	0.36	0.93	0.96	(0.84–1.10)	0.53
rs4939827	18q21.1	*SMAD7*	C	T	0.48	0.85	(0.81–0.89)	1.0 × 10^−12^	Broderick et al. Nat Genet. 2007 (European)	0.78	0.10	0.86	(0.74–1.01)	0.06
rs10411210	19q13.11	*RHPN2*	T	C	0.10	0.87	(0.83–0.91)	4.6 × 10^−09^	Houlston et al. Nat Genet. 2008 (European)	0.21	0.03	0.73	(0.62–0.86)	2.0 × 10^−04^
rs1800469	19q13.2	*B9D2, TGFB1*	G	A	0.48	1.09	(1.06–1.12)	1.2 × 10^−08^	Zhang et al. Nat Genet. 2014 (East Asian)	0.51	0.39	1.04	(0.92–1.19)	0.53
rs2241714	19q13.2	*B9D2, TMEM91*	C	T	0.48	1.09	(1.06–1.12)	1.4 × 10^−08^	Zhang et al. Nat Genet. 2014 (East Asian)	0.51	0.38	1.04	(0.92–1.18)	0.55
rs961253	20p12.3	*Intergenic*	A	C	0.36	1.12	(1.08–1.16)	2.0 × 10^−10^	Houlston et al. Nat Genet. 2008 (European)	0.09	0.97	1.22	(0.99–1.51)	0.07
rs4813802	20p12.3	*Intergenic*	G	T	0.34–0.38	1.09	(1.06–1.12)	7.5 × 10^−11^	Tomlinson et al. PLoS Genet. 2011 (European)	0.21	0.81	1.05	(0.89–1.23)	0.58
rs2423279	20p12.3	*intergenic*	C	T	0.30	1.14	(1.08–1.19)	2.3 × 10^−07^	Jia et al. Nat Genet. 2012 (East Asian)	0.27	0.90	1.19	(1.04–1.37)	0.01

*SNP* single-nucleotide polymorphism, *GWAS* genome-wide association study, *RAF* risk/effect allele frequency, *OR* odds ratio, *CI* confidence interval

^a^Mapped gene was based on the NCBI dbSNP

^b^A1 and A2 were respectively designated as risk/effect and reference allele based on the literature

^c^RAF and *P*_HWE_ were assessed in controls

^d^Unadjusted logistic regression analysis based on additive model

^e^Alleles T instead of A and C instead of G were presented for rs4779584 and rs9929218 in the current study

The G × Es between 31 SNPs and 13 environmental factors were tested using both case-only (Additional file 1: Table S1) and case-control designs (Additional file 1: Table S2). A total of 7 out of 8 G × Es showing the nominal significance of *p*-value < 0.05 in both case-only and case-control analyses satisfied the assumption of independence between genetic and environmental factors except 1 G × E between rs1957636 and smoking status (Additional file 1: Table S3). Table 3 summarizes those 7 G × Es between rs10849432 and BMI, rs11196172 and history of colorectal polyps, rs10795668 and regular exercise, rs4444235 and regular exercise, rs2241714 and regular aspirin use, rs2423279 and regular aspirin use, and rs1957636 and diary consumption in colorectal cancer by study designs. Notably, 2 G × Es between rs4444235 and regular exercise (case-only: *P*_interaction_ = 2.4 × 10^− 3^, case-control: *P*_interaction_ = 1.5 × 10^− 3^) and rs2423279 and regular exercise (case-only: *P*_interaction_ = 7.7 × 10^− 3^, case-control: *P*_interaction_ = 1.6 × 10^− 3^) remained significant in at least one of case-only and case-control analyses even after Bonferroni-corrected *p*-value for multiple testing was allowed for.

**Table 3:** Interactions between susceptibility SNPs and environmental factors in colorectal cancer by study design

Environmental factor	SNP	Chromosomal region	Mapped gene^a^	Allele^b^		Case-only			Case-control		
				A1	A2	OR	(95% CI)^c^	*P*_interaction_^c^	OR	(95% CI)^d^	*P*_interaction_^d^
BMI, kg/m^2^ (≥25 vs. < 25)	rs10849432	12p13.31	intergenic	T	C	1.48	(1.11–1.97)	7.9 × 10^−3^	1.26	(1.04–1.53)	0.02
History of colorectal polyps (yes vs. no)	rs11196172	10q25.2	*TCF7L2*	A	G	2.09	(1.26–3.46)	4.4 × 10^−3^	1.36	(1.01–1.84)	0.05
Regular exercise (no vs. yes)	rs10795668	10p14	*LOC105376400*	A	G	1.42	(1.12–1.81)	4.5 × 10^−3^	1.19	(1.02–1.37)	0.02
Regular exercise (no vs. yes)	rs4444235	14q22.2	intergenic	C	T	1.42	(1.13–1.77)	2.4 × 10^−3^	1.25	(1.09–1.44)	1.5 × 10^−3^
Regular aspirin use (no vs. yes)	rs2241714	19q13.2	*B9D2, TMEM91*	C	T	2.16	(1.08–4.29)	0.03	1.48	(1.04–2.09)	0.03
Regular aspirin use (no vs. yes)	rs2423279	20p12.3	intergenic	C	T	2.46	(1.27–4.75)	7.7 × 10^−3^	1.86	(1.27–2.74)	1.6 × 10^− 3^
Dairy consumption, g/day (≥400 vs. < 400)^e^	rs1957636	14q22.3	*LOC105370507*	A	G	2.27	(1.10–4.69)	0.03	1.60	(1.08–2.35)	0.02

*SNP* single-nucleotide polymorphism, *OR* odds ratio, *CI* confidence interval

^a^Mapped genes were based on the NCBI dbSNP

^b^A1 and A2 were respectively designated as risk/effect and reference allele based on the literature

^c^Logistic regression model based on case-only design using individual SNPs based on additive model and dichotomized environmental factors adjusted age, sex, family history of colorectal cancer, history of DM, regular exercise, and dairy consumption

^d^Logistic regression model based on case-control design using interaction terms including individual SNPs based on additive model and dichotomized environmental facators adjusted age, sex, family history of colorectal cancer, history of DM, regular exercise, and dairy consumption

^e^Dietary factor values were adjusted for total energy intake using the residual method

Table 4 shows the association of 2 SNPs, rs4444235 and rs2423279, with colorectal cancer risk stratified by regular exercise and regular aspirin use. Although the SNP rs4444235 was not significantly associated with colorectal cancer risk (Table 2), the magnitude of the effect of the C allele for this SNP decreased with regular exercise (OR_CC vs. TT_ = 0.58, 95% CI = 0.38–0.88, OR_additive_ = 0.76, 95% CI = 0.62–0.94, OR_recessive_ = 0.66, 95% CI = 0.46–0.94) but increased with nonregular exercise (OR_CC vs. TT_ = 1.47, 95% CI = 1.02–2.10, OR_additive_ = 1.21, 95% CI = 1.01–1.44). The increased magnitude of the effect of the T allele for the SNP rs2423279 on colorectal cancer risk was observed in regular aspirin users (OR_CT vs. CC_ = 4.77, 95% CI = 1.28–17.73, OR_TT vs. CC_ = 21.19, 95% CI = 3.82–117.52, OR_additive_ = 4.62, 95% CI = 1.97–10.80, OR_dominant_ = 6.30, 95% CI = 1.80–22.09, OR_recessive_ = 8.01, 95% CI = 2.02–31.78).

**Table 4:** Associations between susceptibility SNPs and colorectal cancer risk by environmental factors

SNP/genotype^a^	Regular exercise											
	Yes						No					
	Case		Control		OR	(95% CI)^b^	Case		Control		OR	(95% CI)^b^
	N	(%)	N	(%)			N	(%)	N	(%)		
rs4444235 at 14q22.2 (*intergenic*)												
TT	65	(28.4)	185	(22.2)	1.00	(ref.)	98	(20.7)	142	(25.1)	1.00	(ref.)
TC	113	(49.3)	411	(49.3)	0.82	(0.57–1.17)	227	(47.9)	272	(48.1)	1.28	(0.92–1.78)
CC	51	(22.3)	237	(28.5)	0.58	(0.38–0.88)	149	(31.4)	151	(26.7)	1.47	(1.02–2.10)
Additive model					0.76	(0.62–0.94)					1.21	(1.01–1.44)
Dominant model					0.72	(0.52–1.02)					1.35	(0.99–1.83)
Recessive model					0.66	(0.46–0.94)					1.24	(0.94–1.64)
Interaction between rs4444235 and regular exercise												
Case-only^c^	*P* for interaction = 2.4 × 10^−3^											
Case-control^d^	*P* for interaction = 1.5 × 10^− 3^											
rs2423279 at 20p12.3 (*intergenic*)												
CC	5	(25.0)	75	(56.0)	1.00	(ref.)	337	(49.8)	679	(53.5)	1.00	(ref.)
CT	10	(50.0)	53	(39.6)	4.77	(1.28–17.73)	278	(41.1)	498	(39.2)	1.07	(0.87–1.33)
TT	5	(25.0)	6	(4.5)	21.19	(3.82–117.52)	62	(9.2)	93	(7.3)	1.27	(0.87–1.85)
Additive model					4.62	(1.97–10.80)					1.10	(0.94–1.29)
Dominant model					6.30	(1.80–22.09)					1.11	(0.90–1.35)
Recessive model					8.01	(2.02–31.78)					1.23	(0.86–1.77)
Interaction between rs2423279 and regular aspirin use												
Case-only^c^	*P* for interaction = 7.7 × 10^−3^											
Case-control^d^	*P* for interaction = 1.6 × 10^− 3^											

*SNP* single-nucleotide polymorphism, *OR* odds ratio, *CI* confidence interval, *DM* diabetes mellitus

^a^Risk/effect and reference allele was designated based on the literature

^b^Logistic regression modeladjusted age, sex, family history of colorectal cancer, history of DM, regular exercise, and dairy consumption

^c^Logistic regression model based on case-only design using individual SNPs based on additive model and dichotomized environmental facators adjusted age, sex, family history of colorectal cancer, history of DM, regular exercise, and dairy consumption

^d^Logistic regression model based on case-control design using interaction terms including individual SNPs based on additive model and dichotomized environmental facators adjusted age, sex, family history of colorectal cancer, history of DM, regular exercise, and dairy consumption

## Discussion

We evaluated G × Es on colorectal cancer risk for 31 susceptibility SNPs identified through GWAS with 13 established environmental risk or protective factors using both case-only and case-control study designs. Our analysis showed evidence of G × Es between the SNP rs4444235 at 14q22.2 and regular exercise and the SNP rs2423279 at 20p12.3 and regular aspirin use after accounting for multiple testing. Furthermore, we observed that the associations between rs4444235 and rs2423279 were modified by regular exercise and regular aspirin use.

Among previous G × E studies for colorectal cancer susceptibility loci identified by GWAS, the G × E between rs4444235 and regular exercise for colorectal cancer risk has not been investigated [6–13]. The G × E between rs2423279 and regular aspirin was tested by Kantor et al., but the interaction was not detected with a statistically significant level [7]. Furthermore, previously reported G × Es identified by GWAS for colorectal carcinogenesis were not replicated. This may be due to ethnic differences or limited power to detect interactions. We previously reported on G × Es involving GWAS-identified colorectal cancer susceptibility loci with age at cancer onset [13], smoking [11], and alcohol consumption [12] using a conventional method of detecting interactions. In the current analysis, we combined the case-only, case-control, and control-only study designs, suggesting that the results were more powerful and less biased.

Colorectal cancer susceptibility associated with the SNP rs4444235 was first reported by meta-analysis of two GWAS from individuals of European descent [22]. The association between rs4444235 and colorectal cancer risk was also detected among Caucasian and east Asian patients by a meta-analysis [23]. Although several Asian studies [24–26] as well as the current study did not show a statistical association between rs4444235 and colorectal cancer risk, perhaps due to a small sample size, the direction of the association was consistent, suggesting a potential higher risk associated with the C allele. rs4444235 is located at chromosome 14q22.2 close to the bone morphogenetic protein 4 (BMP4) coding gene. Despite the noncoding risk variant, the C allele of rs4444235 showed significantly increased allele-specific expression of the *BMP4* gene in the colorectal cancer cell line [27]. BMP4 is involved in the transforming growth factor beta (TGFβ) superfamily signaling pathway, contributing to colorectal tumorigenesis [28]. Colorectal tumorigenesis may be inhibited by favorable effects of regular exercise stimulating intestinal peristalsis and maintaining the general metabolic milieu [29].

This association between the SNP rs2423279 and colorectal cancer risk was identified by GWAS in east Asians and replicated in east Asians and European-ancestry populations as well [30]. This study also observed that rs2423279 with the C allele was associated with an increased risk of colorectal cancer in the same direction. The 2,423,279 is located at chromosome 20p12.3 close to *HAO1*, which encodes hydroxy acid oxidase 1, and *PLCB1*, which encodes phospholipase C beta 1. In terms of *HAO1* or *PLBC1* genes, the mechanisms of colorectal carcinogenesis and interaction with aspirin are unknown. However, because aspirin can be used as a ligand and/or transport and absorption facilitators of diverse agents, including RNAi or polynucleotide targeting for inhibition of *HAO1* gene expression [31, 32], there still remains a possibility of indirect G × Es between *HAO1* and aspirin use.

A major strength of this study is that we found novel G × Es for colorectal cancer susceptibility loci between SNP rs4444235 and regular exercise and SNP rs2423279 and regular aspirin use after accounting for multiple testing. However, the calculated power was 32.2% for the G × E between rs4444235 and regular exercise and 53.2% for the G × E between rs2423279 and regular aspirin use considering the case-only analysis with 703 cases. Although we did not obtain enough statistical power to detect weak G × Es due to insufficient sample size, both case-only and case-control analyses were performed to overcome sample size limitations, derive additional power, and ensure general validity. Through additional control-only analysis, the assumption of independence of genetic and environmental factors was tested in the underlying population. Also, because the case-only study design estimated interactions on the multiplicative scale, which could not imply that G × Es biologically cause colorectal cancer, case-control study design validated the biological hypotheses.

One limitation is that we did not include all colorectal susceptibility loci identified by previous GWAS in the analyses. However, our genetic factors included a relatively updated and large number of colorectal cancer susceptibility SNPs compared with previous G × E studies for colorectal cancer. Environmental factors in the analysis were also selected based on the latest evidence for colorectal cancer risk or protective factors. The other limitation is that the biological basis of G × Es for GWAS-identified SNPs remains unclear, because the functional relationship between those SNPs based on the agnostic approach and colorectal cancer risk are not fully understood. Third, the observed G × Es have not been validated in the other population. We further conducted case-only and case-control analysis on G × Es between rs4444235 and regular exercise among Whites in UK Biobank, no statistically significant interactions were observed (Additional file 1: Table S4). Further studies for Asian-based established risk and protective factors on colorectal cancer and validation studies with sufficient sample size are warranted.

## Conclusions

In conclusion, our results suggest that there are possible interactions between the SNP rs4444235 at 14q22.2 and regular exercise and the SNP rs2423279 at 20p12.3 and regular aspirin use in colorectal carcinogenesis.

## Additional files

**Additional file 1 **: **Figure S1.** A flow diagram of the study population. **Table S1.** Interactions between susceptibility SNPs and environmental factors in colorectal cancer by case-only analysis. **Table S2.** Interactions between susceptibility SNPs and environmental factors in colorectal cancer by case-control analysis. **Table S3.** Independence test between selected susceptibility SNPs and environmental factors by control-only analysis. **Table S4.** Associations between rs4444235 and colorectal cancer risk by regular exercise among Whites in UK Biobank.

## Data Availability

The datasets generated during and/or analyzed during the current study are available from the corresponding author on reasonable request.

## Abbreviations

ACS: American Cancer Society
BMP4: Bone morphogenetic protein 4
CI: Confidence interval
G × E: Gene-environment interaction
GWAS: Genome-wide association study
HWE: Hardy-Weinberg equilibrium
IBD: Inflammatory bowel disease
IRB: Institutional review board
MAF: Minor allele frequency
NCC: National Cancer Center
NCI: National Cancer Institute
NRF: National Research Foundation of Korea
OR: Odds ratio
PDQ: Physician Data Query
RAF: Risk allele frequency
SNP: Single-nucleotide polymorphism
SQFFQ: Semiquantitative food frequency questionnaire
TGFβ: Transforming growth factor beta
