# Supplementary material for: Evaluation of gene-environment interactions for colorectal cancer susceptibility loci using case-only and case-control designs
Source: BMC Cancer. 2019 Dec 18;19:1231. doi: 10.1186/s12885-019-6456-9 (PMC6918639; doi:10.1186/s12885-019-6456-9)
Supplement: Supplementary file 1 — Additional file 1 : Figure S1. A flow diagram of the study population. Table S1. Interactions between susceptibility SNPs and environmental factors in colorectal cancer by case-only analysis. Table S2. Interactions between susceptibility SNPs and environmental factors in colorectal cancer by case-control analysis. Table S3. Independence test between selected susceptibility SNPs and environmental factors by control-only analysis. Table S4. Associations between rs4444235 and colorectal cancer risk by regular exercise among Whites in UK Biobank. [file 12885_2019_6456_MOESM1_ESM.docx]

Supplementary Figure 1. A flow diagram of the study population.


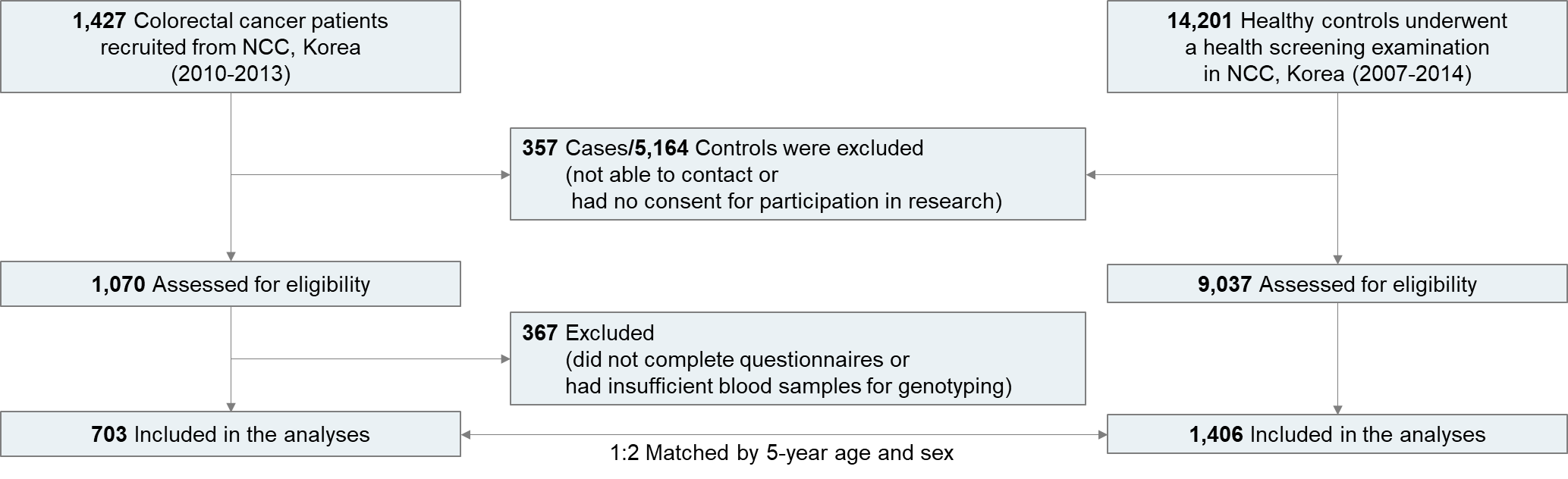


| Supplementary Table 1. Interactions between susceptibility SNPs and environmental factors in colorectal cancer by case-only analysis | | | | | | | | | | | | | | | | | | | | | | | | | | | | | |
| --- | --- | --- | --- | --- | --- | --- | --- | --- | --- | --- | --- | --- | --- | --- | --- | --- | --- | --- | --- | --- | --- | --- | --- | --- | --- | --- | --- | --- | --- |
| SNP | Chromosomal  region | Mapped gene^†^ | Allele^‡^ | | *P* for interaction by case-only analysis^§^ | | | | | | | | | | | | | | | | | | | | | | | | |
|  |  |  | A1 | A2 | E1 | | E2 | | E3 | | E4 | | E5 | | E6 | | E7 | | E8 | | E9 | | E10 | | E11 | | E12 | | E13 |
| rs6687758 | 1q41 | *intergenic* | G | A | 0.22 | | 0.42 | | 0.14 | | 0.09 | | 0.79 | | 0.62 | | 0.37 | | 0.05 | | 0.21 | | 0.36 | | 0.38 | | 0.91 | | 0.49 |
| rs10936599 | 3q26.2 | *MYNN* | T | C | 0.55 | | 0.43 | | 0.05 | | 0.50 | | 0.50 | | 0.91 | | 0.70 | | 0.63 | | 0.25 | | 0.52 | | 0.93 | | 0.92 | | 0.75 |
| rs647161 | 5q31.1 | *C5orf66* | A | C | 0.42 | | 0.22 | | 0.17 | | 0.75 | | 0.54 | | 0.97 | | 0.88 | | 0.82 | | 0.07 | | 0.76 | | 0.19 | | 0.62 | | 0.40 |
| rs7758229 | 6q25.3 | *SLC22A3* | T | G | 0.65 | | 0.96 | | 0.83 | | 0.88 | | 0.47 | | 0.81 | | 0.74 | | 0.57 | | 0.75 | | 0.94 | | 0.30 | | 0.81 | | 0.79 |
| rs6983267 | 8q24.21 | *CASC8, CCAT2* | G | T | 0.38 | | 0.81 | | 0.64 | | 0.89 | | 0.50 | | 0.52 | | 0.87 | | 0.09 | | 0.50 | | 0.46 | | 0.76 | | 0.97 | | 0.96 |
| rs7014346 | 8q24.21 | *CASC8* | A | G | 0.88 | | 0.73 | | 0.85 | | 0.79 | | 0.79 | | 0.10 | | 0.97 | | 0.06 | | 0.59 | | 0.73 | | 0.52 | | 0.60 | | 0.89 |
| rs10505477 | 8q24.21 | *CASC8* | A | G | 0.53 | | 0.85 | | 0.48 | | 0.89 | | 0.61 | | 0.50 | | 0.99 | | 0.13 | | 0.62 | | 0.44 | | 0.98 | | 0.96 | | 0.68 |
| rs10795668 | 10p14 | *LOC105376400* | A | G | 0.16 | | 0.15 | | 0.84 | | 0.94 | | 0.90 | | 0.94 | | 0.85 | | 4.5×10^-3^ | | 0.74 | | 0.35 | | 0.15 | | 0.53 | | 0.77 |
| rs704017 | 10q22.3 | *ZMIZ1-AS1* | G | A | 0.30 | | 0.73 | | 0.91 | | 0.12 | | 0.14 | | 0.20 | | 0.69 | | 0.99 | | 0.62 | | 0.78 | | 0.41 | | 0.86 | | 0.27 |
| rs11196172 | 10q25.2 | *TCF7L2* | A | G | 0.03 | | 0.54 | | 4.4×10^-3^ | | 0.92 | | 0.84 | | 0.81 | | 0.95 | | 0.59 | | 0.86 | | 0.95 | | 0.31 | | 0.94 | | 0.43 |
| rs1665650 | 10q26.2 | *HSPA12A* | T | C | 0.20 | | 0.87 | | 0.11 | | 0.42 | | 0.26 | | 0.45 | | 0.51 | | 0.75 | | 0.40 | | 0.33 | | 0.88 | | 0.57 | | 0.46 |
| rs174537 | 11q12.2 | *MYRF* | G | T | 0.93 | | 0.28 | | 0.84 | | 0.89 | | 0.72 | | 0.09 | | 0.50 | | 0.21 | | 0.97 | | 0.60 | | 0.80 | | 0.24 | | 0.77 |
| rs174550 | 11q12.2 | *FADS1* | T | C | 0.92 | | 0.28 | | 0.81 | | 0.86 | | 0.79 | | 0.07 | | 0.39 | | 0.11 | | 0.75 | | 0.65 | | 0.55 | | 0.24 | | 0.54 |
| rs1535 | 11q12.2 | *FADS2* | A | G | 0.95 | | 0.32 | | 0.67 | | 0.87 | | 0.71 | | 0.07 | | 0.45 | | 0.15 | | 0.89 | | 0.65 | | 0.72 | | 0.24 | | 0.51 |
| rs3802842 | 11q23.1 | *COLCA1, COLCA2* | C | A | 0.65 | | 1.00 | | 0.75 | | 0.94 | | 0.19 | | 0.45 | | 0.05 | | 0.77 | | 0.53 | | 0.11 | | 0.79 | | 0.82 | | 0.17 |
| rs10849432 | 12p13.31 | *intergenic* | T | C | 7.9×10^-3^ | | 0.21 | | 0.61 | | 0.96 | | 0.72 | | 0.12 | | 0.42 | | 0.96 | | 0.36 | | 0.91 | | 0.07 | | 0.96 | | 0.21 |
| rs10774214 | 12p13.32 | *CCND2-AS1* | T | C | 0.22 | | 0.46 | | 0.09 | | 0.44 | | 0.18 | | 0.52 | | 0.29 | | 0.69 | | 0.52 | | 0.74 | | 0.57 | | 0.22 | | 0.07 |
| rs11169552 | 12q13.13 | *ATF1, LOC105369765* | T | C | 0.49 | | 0.25 | | 0.09 | | 0.94 | | 0.37 | | 0.64 | | 0.22 | | 0.46 | | 0.66 | | 0.84 | | 0.90 | | 0.16 | | 0.06 |
| rs7136702 | 12q13.13 | *intergenic* | T | C | 0.74 | | 0.95 | | 0.35 | | 0.36 | | 0.61 | | 0.69 | | 0.08 | | 0.41 | | 0.63 | | 0.21 | | 0.78 | | 0.43 | | 0.18 |
| rs4444235 | 14q22.2 | *intergenic* | C | T | 0.86 | | 0.87 | | 0.09 | | 0.88 | | 0.71 | | 0.27 | | 0.89 | | 2.4×10^-3^ | | 0.20 | | 0.99 | | 0.25 | | 0.34 | | 0.35 |
| rs1957636 | 14q22.3 | *LOC105370507* | A | G | 0.28 | | 0.27 | | 0.84 | | 0.55 | | 0.44 | | 0.16 | | 2.9×10^-3^ | | 0.39 | | 0.08 | | 0.35 | | 0.61 | | 0.71 | | 0.03 |
| rs4779584 | 15q13.3 | *intergenic* | T | C | 0.84 | | 0.96 | | 0.89 | | 0.95 | | 0.54 | | 0.85 | | 0.43 | | 0.48 | | 0.53 | | 0.32 | | 0.37 | | 0.96 | | 0.89 |
| rs9929218 | 16q22.1 | *CDH1* | T | C | 0.62 | | 0.99 | | 0.61 | | 0.59 | | 0.41 | | 0.50 | | 0.95 | | 0.99 | | 0.64 | | 0.03 | | 0.48 | | 0.47 | | 0.11 |
| rs12603526 | 17p13.3 | *NXN* | C | T | 0.64 | | 0.19 | | 0.65 | | 0.59 | | 0.36 | | 0.06 | | 0.07 | | 0.52 | | 0.28 | | 0.24 | | 0.58 | | 0.31 | | 0.23 |
| rs4939827 | 18q21.1 | *SMAD7* | C | T | 0.37 | | 0.02 | | 0.52 | | 0.99 | | 0.47 | | 0.61 | | 0.50 | | 0.14 | | 0.90 | | 0.60 | | 0.97 | | 0.94 | | 0.29 |
| rs10411210 | 19q13.11 | *RHPN2* | T | C | 0.10 | | 0.34 | | 0.13 | | 0.52 | | 0.95 | | 0.63 | | 0.60 | | 0.95 | | 0.47 | | 0.18 | | 1.1×10^-3^ | | 0.24 | | 0.21 |
| rs1800469 | 19q13.2 | *B9D2, TGFB1* | G | A | 0.65 | | 0.50 | | 0.65 | | 0.43 | | 0.52 | | 0.90 | | 0.44 | | 0.65 | | 0.06 | | 0.16 | | 0.69 | | 0.25 | | 0.75 |
| rs2241714 | 19q13.2 | *B9D2, TMEM91* | C | T | 0.88 | | 0.39 | | 0.99 | | 0.43 | | 0.44 | | 0.78 | | 0.35 | | 0.54 | | 0.03 | | 0.31 | | 0.42 | | 0.25 | | 0.78 |
| rs961253 | 20p12.3 | *intergenic* | A | C | 0.64 | | 0.39 | | 0.38 | | 0.95 | | 0.27 | | 0.59 | | 0.46 | | 0.80 | | 0.40 | | 0.25 | | 0.95 | | 0.29 | | 0.85 |
| rs4813802 | 20p12.3 | *intergenic* | G | T | 0.91 | | 0.81 | | 0.27 | | 0.85 | | 4.6×10^-3^ | | 0.29 | | 0.16 | | 1.00 | | 0.88 | | 0.77 | | 0.82 | | 0.94 | | 0.66 |
| rs2423279 | 20p12.3 | *intergenic* | C | T | 0.86 | | 0.17 | | 0.24 | | 0.41 | | 0.70 | | 0.33 | | 0.36 | | 0.25 | | 7.7×10^-3^ | | 0.40 | | 0.83 | | 0.90 | | 0.31 |
| Abbrevations: SNP (single-nucleotide polymorphism), BMI (body mass index), IBD (inflammatory bowel disease), DM (diabetes mellitus), and HRT (hormone replace therapy). | | | | | | | | | | | | | | | | | | | | | | | | | | | | | |
| ^†^Mapped genes were based on the NCBI dbSNP. | | |  |  |  |  | |  | |  | |  | |  | |  | |  | |  | |  | |  | |  | |  | |
| ^‡^A1 and A2 were respectively designated as risk/effect and reference allele based on the literature. | | | | | | | | | | | | | |  | |  | |  | |  | |  | |  | |  | |  | |
| ^§^Logistic regression analysis using individual SNPs based on additive model as independent variables and dichotomized environmental factors as outcome variables (E1: BMI, E2: family history of colorectal cancer, E3: history of colorectal polyps, E4 history of IBD, E5: history of DM, E6: alcohol drinking, E7: smoking, E8: regular exercise, E9: regular aspirin use, E10: HRT in postmenopausal women, E11: red meat intake, E12: processed meat intake, E13: dairy consumption) adjusted age, sex, family history of colorectal cancer, history of DM, regular exercise, and dairy consumption. | | | | | | | | | | | | | | | | | | | | | | | | | | | | | |

| Supplementary Table 2. Interactions between susceptibility SNPs and environmental factors in colorectal cancer by case-control analysis | | | | | | | | | | | | | | | | | | | | | | | | | | | |
| --- | --- | --- | --- | --- | --- | --- | --- | --- | --- | --- | --- | --- | --- | --- | --- | --- | --- | --- | --- | --- | --- | --- | --- | --- | --- | --- | --- |
| SNP | Chromosomal  region | Mapped gene^†^ | Allele^‡^ | | *P* for interaction by case-only analysis^§^ | | | | | | | | | | | | | | | | | | | | | | |
|  |  |  | A1 | A2 | E1 | E2 | E3 | | E4 | | E5 | | E6 | | E7 | | E8 | | E9 | | E10 | E11 | | E12 | | E13 | |
| rs6687758 | 1q41 | *intergenic* | G | A | 0.50 | 0.18 | 0.58 | | 0.97 | | 0.94 | | 0.03 | | 0.11 | | 0.25 | | 0.59 | | 0.04 | 0.16 | | 0.97 | | 0.57 | |
| rs10936599 | 3q26.2 | *MYNN* | T | C | 0.86 | 0.41 | 0.31 | | 0.67 | | 0.50 | | 0.76 | | 0.79 | | 0.16 | | 0.08 | | 0.63 | 0.33 | | 0.97 | | 0.78 | |
| rs647161 | 5q31.1 | *C5orf66* | A | C | 0.24 | 0.70 | 0.33 | | 0.47 | | 0.94 | | 0.55 | | 0.72 | | 0.06 | | 0.03 | | 0.80 | 0.24 | | 0.86 | | 0.60 | |
| rs7758229 | 6q25.3 | *SLC22A3* | T | G | 0.49 | 0.70 | 0.47 | | 0.75 | | 0.31 | | 0.35 | | 0.35 | | 0.36 | | 0.26 | | 0.57 | 0.46 | | 0.21 | | 0.96 | |
| rs6983267 | 8q24.21 | *CASC8, CCAT2* | G | T | 0.32 | 0.59 | 0.55 | | 0.53 | | 0.63 | | 0.44 | | 0.93 | | 0.02 | | 0.33 | | 0.28 | 0.90 | | 0.93 | | 0.98 | |
| rs7014346 | 8q24.21 | *CASC8* | A | G | 0.48 | 0.77 | 0.43 | | 0.37 | | 0.86 | | 0.44 | | 0.36 | | 0.04 | | 0.22 | | 0.95 | 0.40 | | 0.90 | | 0.90 | |
| rs10505477 | 8q24.21 | *CASC8* | A | G | 0.46 | 0.58 | 0.46 | | 0.52 | | 0.77 | | 0.59 | | 0.69 | | 0.03 | | 0.47 | | 0.35 | 0.61 | | 0.76 | | 0.76 | |
| rs10795668 | 10p14 | *LOC105376400* | A | G | 0.19 | 0.13 | 0.72 | | 0.96 | | 0.35 | | 0.92 | | 0.73 | | 0.02 | | 0.90 | | 0.34 | 0.30 | | 0.99 | | 0.73 | |
| rs704017 | 10q22.3 | *ZMIZ1-AS1* | G | A | 0.70 | 0.99 | 0.54 | | 0.96 | | 0.73 | | 0.63 | | 0.95 | | 0.19 | | 0.31 | | 0.72 | 0.24 | | 0.96 | | 0.71 | |
| rs11196172 | 10q25.2 | *TCF7L2* | A | G | 0.20 | 0.39 | 0.05 | | 0.67 | | 0.86 | | 0.30 | | 0.93 | | 0.28 | | 0.81 | | 0.64 | 0.38 | | 0.96 | | 0.12 | |
| rs1665650 | 10q26.2 | *HSPA12A* | T | C | 5.2×10^-3^ | 0.62 | 0.16 | | 0.97 | | 0.18 | | 0.15 | | 0.14 | | 0.54 | | 0.87 | | 0.11 | 0.75 | | 0.68 | | 0.33 | |
| rs174537 | 11q12.2 | *MYRF* | G | T | 0.91 | 0.41 | 0.43 | | 0.63 | | 0.69 | | 0.98 | | 0.21 | | 0.33 | | 0.94 | | 0.51 | 0.17 | | 0.97 | | 0.77 | |
| rs174550 | 11q12.2 | *FADS1* | T | C | 0.90 | 0.32 | 0.43 | | 0.65 | | 0.76 | | 1.00 | | 0.31 | | 0.20 | | 0.63 | | 0.52 | 0.23 | | 0.59 | | 0.60 | |
| rs1535 | 11q12.2 | *FADS2* | A | G | 0.82 | 0.33 | 0.52 | | 0.64 | | 0.78 | | 0.91 | | 0.24 | | 0.27 | | 0.94 | | 0.54 | 0.20 | | 0.97 | | 0.56 | |
| rs3802842 | 11q23.1 | *COLCA1, COLCA2* | C | A | 0.80 | 0.02 | 0.42 | | 0.93 | | 0.34 | | 0.06 | | 0.11 | | 0.88 | | 0.37 | | 0.07 | 0.40 | | 0.29 | | 0.16 | |
| rs10849432 | 12p13.31 | *intergenic* | T | C | 0.02 | 0.31 | 0.95 | | 0.97 | | 0.11 | | 0.05 | | 0.96 | | 0.82 | | 0.77 | | 0.81 | 0.34 | | - | | 0.19 | |
| rs10774214 | 12p13.32 | *CCND2-AS1* | T | C | 0.46 | 0.20 | 0.04 | | 0.96 | | 0.77 | | 0.07 | | 0.97 | | 0.15 | | 0.07 | | 0.60 | 0.29 | | 0.97 | | 0.22 | |
| rs11169552 | 12q13.13 | *ATF1, LOC105369765* | T | C | 0.30 | 0.98 | 0.22 | | 0.96 | | 0.65 | | 0.84 | | 0.80 | | 0.69 | | 0.77 | | 0.68 | 0.73 | | 0.43 | | 0.03 | |
| rs7136702 | 12q13.13 | *intergenic* | T | C | 0.40 | 0.85 | 0.55 | | 0.34 | | 0.68 | | 0.87 | | 0.95 | | 0.47 | | 0.20 | | 0.16 | 0.91 | | 0.54 | | 0.13 | |
| rs4444235 | 14q22.2 | *intergenic* | C | T | 0.25 | 0.66 | 1.4×10^-3^ | | 0.76 | | 0.48 | | 0.76 | | 0.14 | | 1.5×10^-3^ | | 0.13 | | 0.39 | 0.88 | | 0.91 | | 0.28 | |
| rs1957636 | 14q22.3 | *LOC105370507* | A | G | 0.23 | 0.74 | 0.93 | | 0.97 | | 0.27 | | 0.27 | | 0.01 | | 0.08 | | 0.03 | | 0.54 | 0.39 | | 0.24 | | 0.02 | |
| rs4779584 | 15q13.3 | *intergenic* | T | C | 0.93 | 0.71 | 0.36 | | 0.61 | | 0.42 | | 0.85 | | 0.34 | | 0.86 | | 0.99 | | 0.11 | 0.20 | | 0.98 | | 0.92 | |
| rs9929218 | 16q22.1 | *CDH1* | T | C | 0.75 | 0.24 | 0.79 | | 0.60 | | 0.50 | | 0.40 | | 0.72 | | 0.93 | | 0.44 | | 0.08 | 0.09 | | 0.70 | | 0.06 | |
| rs12603526 | 17p13.3 | *NXN* | C | T | 0.87 | 0.81 | 0.27 | | 0.48 | | 0.16 | | 0.51 | | 0.41 | | 0.61 | | 0.48 | | 0.41 | 0.42 | | 0.98 | | 0.22 | |
| rs4939827 | 18q21.1 | *SMAD7* | C | T | 0.18 | 0.31 | 0.73 | | 0.29 | | 0.93 | | 0.46 | | 0.71 | | 0.17 | | 0.77 | | 0.53 | 0.32 | | 0.97 | | 0.39 | |
| rs10411210 | 19q13.11 | *RHPN2* | T | C | 0.24 | 0.17 | 0.27 | | 0.74 | | 0.27 | | 0.56 | | 0.80 | | 0.55 | | 0.86 | | 0.81 | 0.05 | | 0.85 | | 0.70 | |
| rs1800469 | 19q13.2 | *B9D2, TGFB1* | G | A | 0.06 | 0.77 | 0.73 | | 0.97 | | 0.84 | | 0.72 | | 0.81 | | 0.36 | | 0.08 | | 0.63 | 0.54 | | 0.46 | | 0.85 | |
| rs2241714 | 19q13.2 | *B9D2, TMEM91* | C | T | 0.37 | 0.82 | 0.72 | | 0.66 | | 0.39 | | 0.69 | | 0.80 | | 0.96 | | 0.03 | | 0.24 | 0.21 | | 0.96 | | 0.91 | |
| rs961253 | 20p12.3 | *intergenic* | A | C | 0.33 | 0.58 | 0.23 | | 0.03 | | 0.84 | | 0.70 | | 0.85 | | 0.47 | | 0.42 | | 0.50 | 0.58 | | 0.97 | | 0.69 | |
| rs4813802 | 20p12.3 | *intergenic* | G | T | 0.90 | 0.77 | 0.27 | | 0.64 | | 0.07 | | 0.09 | | 0.10 | | 0.58 | | 0.78 | | 0.77 | 0.31 | | - | | 0.90 | |
| rs2423279 | 20p12.3 | *intergenic* | C | T | 0.50 | 0.37 | 0.25 | | 0.97 | | 0.90 | | 0.89 | | 0.92 | | 0.15 | | 1.6×10^-3^ | | 1.00 | 0.26 | | 0.71 | | 0.38 | |
| ^†^Mapped genes were based on the NCBI dbSNP. | | |  |  |  |  | |  | |  | |  | |  | |  | |  | |  |  | |  | |  | |  |
| ^‡^A1 and A2 were respectively designated as risk/effect and reference allele based on the literature. | | | | | | | | | | | | | |  | |  | |  | |  |  | |  | |  | |  |
| ^§^Logistic regression model based on case-control design using interaction terms including individual SNPs based on additive model and dichotomized environmental factors (E1: BMI, E2: family history of colorectal cancer, E3: history of colorectal polyps, E4: history of IBD, E5: history of DM, E6: alcohol drinking, E7: smoking, E8: regular exercise, E9: regular aspirin use, E10: HRT in postmenopausal women, E11: red meat intake, E12: processed meat intake, E13: dairy consumption) adjusted age, sex, family history of colorectal cancer, history of DM, regular exercise, and dairy consumption. | | | | | | | | | | | | | | | | | | | | | | | | | | | |

| Supplementary Table 3. Independence test between selected susceptibility SNPs and environmental factors by control-only analysis | | | | | | | | |
| --- | --- | --- | --- | --- | --- | --- | --- | --- |
| Environmental factor | SNP | Chromosomal  region | Mapped gene^†^ | Allele^‡^ | | Control-only | | |
|  |  |  |  | A1 | A2 | OR | (95% CI) ^§^ | *P*^§^ |
| BMI, kg/m^2^ (≥25 vs. <25) | rs10849432 | 12p13.31 | *intergenic* | T | C | 0.99 | (0.80-1.22) | 0.94 |
| History of colorectal polyps (yes vs. no) | rs11196172 | 10q25.2 | *TCF7L2* | A | G | 1.10 | (0.84-1.43) | 0.49 |
| Smoking (ever vs. never) | rs1957636 | 14q22.3 | *LOC105370507* | A | G | 1.35 | (1.08-1.67) | 0.01 |
| Regular exercise (no vs. yes) | rs10795668 | 10p14 | *LOC105376400* | A | G | 1.00 | (0.85-1.17) | 0.96 |
| Regular exercise (no vs. yes) | rs4444235 | 14q22.2 | *intergenic* | C | T | 0.89 | (0.77-1.04) | 0.15 |
| Regular aspirin use (yes vs. no) | rs2241714 | 19q13.2 | *B9D2, TMEM91* | C | T | 0.98 | (0.75-1.27) | 0.85 |
| Regular aspirin use (yes vs. no) | rs2423279 | 20p12.3 | *intergenic* | C | T | 0.85 | (0.62-1.16) | 0.30 |
| Dairy consumption, g/day (≥400 vs. <400) ^¶^ | rs1957636 | 14q22.3 | *LOC105370507* | A | G | 0.92 | (0.74-1.15) | 0.45 |
| Abbrevations: SNP (single-nucleotide polymorphism), OR (odds ratio), and CI (confidence interval). | | | | |  |  |  |  |
| ^†^Mapped genes were based on the NCBI dbSNP. | |  |  |  |  |  |  |  |
| ^‡^A1 and A2 were respectively designated as risk/effect and reference allele based on the literature. | | | | |  |  |  |  |
| ^§^Logistic regression model based on control-only design using individual SNPs based on additive model and dichotomized environmental factors adjusted age, sex, family history of colorectal cancer, history of DM, regular exercise, and dairy consumption. | | | | | | | | |
| ^¶^Dietary factor values were adjusted for total energy intake using the residual method. | | | | | | | | |

| Supplementary Table 4. Associations between rs4444235 and colorectal cancer risk by regular exercise among Whites in UK Biobank | | | | | | | | | | | | |
| --- | --- | --- | --- | --- | --- | --- | --- | --- | --- | --- | --- | --- |
| SNP/genotype^a^ | Regular exercise | | | | | | | | | | | |
|  | Yes | | | | | | No | | | | | |
|  | Case | | Control | | OR | (95% CI)^b^ | Case | | Control | | OR | (95% CI)^b^ |
|  | N | (%) | N | (%) |  |  | N | (%) | N | (%) |  |  |
| rs4444235 at 14q22.2 (*intergenic*) among Whites | | | | |  |  |  |  |  |  |  |  |
| TT | 694 | (26.4) | 1,603 | (28.8) | 1.00 | (ref.) | 452 | (27.4) | 863 | (28.3) | 1.00 | (ref.) |
| TC | 1,310 | (49.9) | 2,737 | (49.1) | 1.11 | (0.99-1.24) | 810 | (49.1) | 1,547 | (50.8) | 1.01 | (0.87-1.16) |
| CC | 611 | (23.3) | 1,200 | (21.5) | 1.18 | (1.04-1.35) | 378 | (22.9) | 618 | (20.3) | 1.17 | (0.99-1.39) |
| Additive model | |  |  |  | 1.09 | (1.02-1.16) | |  |  |  | 1.08 | (0.99-1.17) |
| Dominant model | |  |  |  | 1.13 | (1.02-1.26) | |  |  |  | 1.05 | (0.92-1.20) |
| Recessive model | |  |  |  | 1.11 | (0.99-1.24) | |  |  |  | 1.17 | (1.01-1.35) |
| Interaction between rs4444235 and regular exercise | | | | | | | | | | | | |
| Case-only^c^ | *P* for interaction=0.52 | | | | | | | | | | | |
| Case-control^d^ | *P* for interaction=0.84 | | | | | | | | | | | |
| Abbrevations: SNP (single-nucleotide polymorphism), OR (odds ratio), and CI (confidence interval). | | | | | | | | | | | | |
| ^a^Risk/effect and reference allele was designated based on the literature. | | | | | | | | | | | | |
| ^b^Logistic regression modeladjusted age and sex. | | | | | | | | | | | | |
| ^c^Logistic regression model based on case-only design using individual SNPs based on additive model and dichotomized environmental factors adjusted age and sex. | | | | | | | | | | | | |
| ^d^Logistic regression model based on case-control design using interaction terms including individual SNPs based on additive model and dichotomized environmental facators adjusted age and sex. | | | | | | | | | | | | |
